# Supplementary material for: Conditional chemoconnectomics (cCCTomics) as a strategy for efficient and conditional targeting of chemical transmission
Source: eLife. 2024 Apr 30;12:RP91927. doi: 10.7554/eLife.91927 (PMC11060718; doi:10.7554/eLife.91927)
Supplement: Figure 3—source data 3. [file elife-91927-fig3-data3.zip › Figure 3 source data3 part 1.zip!/Figure 3-Source data 3-part1/1063-13-CRISPResso_Report_8cz_fsdr.zip!/CRISPResso_on_1063_16/2a.Nucleotide_percentage_quilt.pdf]

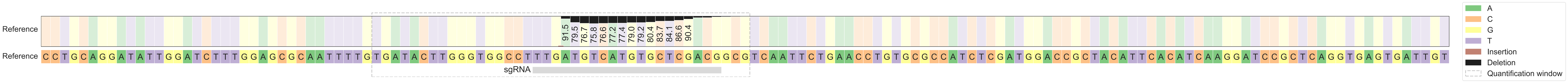

Reference
91.5
79.5
76.7
75.8
76.6
77.2
77.4
79.0
79.2
80.4
83.7
84.1
86.6
90.4
Reference
CCTGCAGGATATTGGATCTTTGGAGCGCAATTTTGTGATACTTGGGTGGCCTTTGATGTCATGTGCTCGACGGCGTCAATTCTGAACCTGTGCGCCATCTCGATGGACCGCTACATTCACATCAAGGATCCGCTCAGGTGAGTGATTGT
sgRNA
A
C
G
T
Insertion
Deletion
Quantification window
